# Supplementary material for: N-Butyldeoxygalactonojirimycin Induces Reversible Infertility in Male CD Rats
Source: Int J Mol Sci. 2019 Dec 31;21(1):301. doi: 10.3390/ijms21010301 (PMC6982022; doi:10.3390/ijms21010301)
Supplement: Supplementary file 1 [file ijms-21-00301-s001.pdf]

International Journal of *Molecular Sciences*

MDPI

Article (Supporting Information)

N-Butyldeoxygalactonojirimycin induces reversible infertility in male CD rats

Vijayalaxmi Gupta,[1a] Sheri A. Hild,[2#] Sudhakar R. Jakkaraj,[3] Erick J. Carlson,[3] Henry L. Wong,[3] Gunda I. Georg,[3] and Joseph S. Tash[1*]

[1]Department of Molecular and Integrative Physiology, University of Kansas Medical Center, Kansas City, Kansas, United States of America

[2]Division of Reproductive Endocrinology and Toxicology, BIOQUAL, Inc., Rockville, Maryland, United States of America

[3]Department of Medicinal Chemistry, and Institute for Therapeutics Discovery and Development, College of Pharmacy, University of Minnesota, Minneapolis, Minnesota, United States of America

Current address:

[#]Division of Comparative Medicine, ORIP, DPCPSI, Bethesda, Maryland, United States of America

[a]Department of Obstetrics and Gynecology, University of Kansas Medical Center, Kansas City, Kansas, United States of America

[*]**Corresponding author,** E-mail: jtash@kumc.edu (JT)

Received: date; Accepted: date; Published: date

Contents:

Synthesis of *N*-butyl-1-deoxynojirimycin (*N*B-DNJ) S3

Synthesis of *N*-butyl-1-deoxygalactonojirimycin (*N*B-DGJ) S5

Purity determination by LC/MS S5

LC/MS traces for NB-DNJ S7

LC/MS traces for NB-DGJ S8

Figure showing hormone levels in rats during *N*B-DGJ treatment schedule S9

Figure showing rat epididymal sperm motility S10

Figure showing rat epididymal sperm morphology S10

Figure showing study design S10

References S10

Chemistry:

General methods. Unless specified, all reactions were performed under a nitrogen atmosphere in oven-dried glassware. Solvents were dried before use over an activated alumina column. All commercial reagents were used as received. NMR data were recorded using a 400/100 MHz or a 500/125 MHz spectrometer.

***N*-Butyl-1-deoxynojirimycin (*N*B-DNJ).** The synthesis of *N*-butyl-1-deoxynojirimycin (*N*B-DNJ) was accomplished following the procedures of Matos et al. (Matos et al. 1999) and Amann et al. as shown in Scheme 1.[1,2] 2,3,4,6-Tetra-*O*-benzyl-α-glucopyranose was reduced with lithium aluminum hydride to furnish 2,3,4,6-tetra-*O*-benzyl-D-sorbitol (**1**). Intermediate **1** was oxidized to ketoaldehyde **2** and subjected to reductive amination with n-butylamine (n-BuNH_2) and sodium cyanoborohydride to provide 2,3,4,6-tetra-*O*-benzyl-*N*-butyl-1,5-dideoxy-1,5-D-glucitol (**3**). Hydrogenolysis of **3** yielded the target compound *N*-butyl-1-deoxynojirimycin (*N*B-DNJ). *N*-Butyl-1-deoxygalactonojirimycin (*N*B-DGJ) was prepared following the same procedure using 2,3,4,6-tetra-*O*-benzyl-D-glactonopyranose as the starting material. The spectroscopic data obtained for *N*B-DNJ and *N*B-DGJ matched the literature values.[1,2]

Scheme 1. Synthesis of *N*-butyl-1-deoxynojirimycin (*N*B-DNJ).

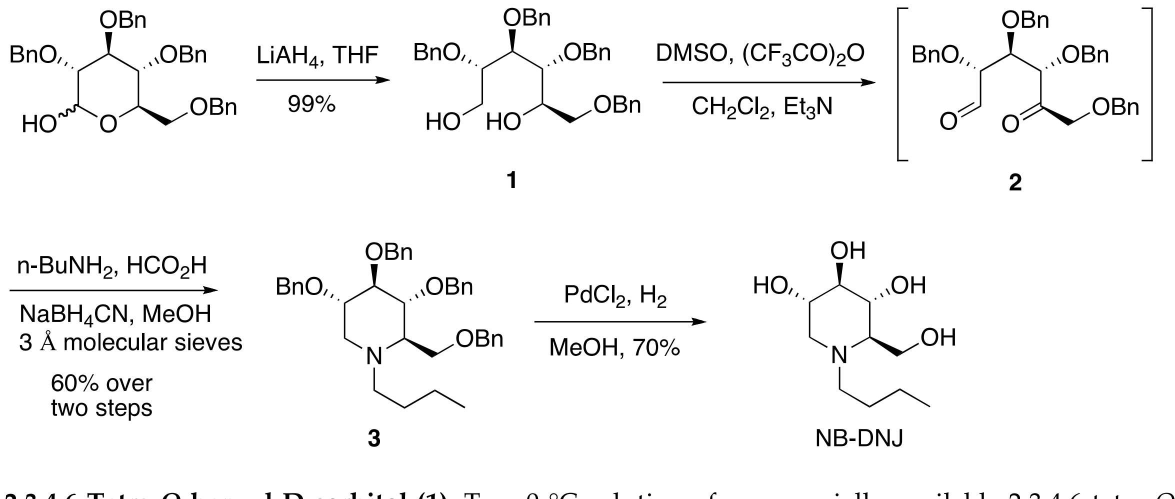

2,3,4,6-Tetra-*O*-benzyl-D-sorbitol (1). To a 0 °C solution of commercially available 2,3,4,6-tetra-*O*-benzyl-α-glucopyranose (5.00 g, 9.24 mmol) in anhydrous THF (100 mL), $LiAlH_4$ (1.20 g, 31.6 mmol, 3.43 equiv) was added carefully in small portions. The mixture was stirred overnight at room temperature and then cooled to 0 °C. After the excess of $LiAlH_4$ was destroyed by the careful addition of ethyl acetate (20 mL), additional ethyl acetate (500 mL) was added. Then 2 N aq HCl

(250 mL) was added and the reaction mixture was stirred for 10 min. The organic layer was separated, washed successively with sat. aq. $NaHCO_3$ (150 mL), dried with Na_2SO_4, and evaporated affording 5.0 g (99%) of **1** as a colorless viscous syrup.

2,3,4,6-Tetra-*O*-benzyl-*N*-butyl-1,5-dideoxy-1,5-D-glucitol (3). To a –78 °C mixture of dry CH_2Cl_2 (25 mL) and anhydrous DMSO (4.46 g, 4.05 mL, 57.1 mmol, 6.2 equiv) under an inert gas atmosphere was added dropwise a solution of trifluoroacetic anhydride (8.87 g, 5.87 mL, 42.2 mmol, 4.5 equiv) in CH_2Cl_2 (25 mL). After the mixture was stirred for 1.5h at –78 °C, a solution of **1** (5.00 g, 9.21 mmol) in CH_2Cl_2 (50 mL) was added dropwise while maintaining the temperature of the reaction mixture below –78 °C during the addition. The mixture was stirred for an additional 2h at –78°C and then a solution of Et_3N (7.52 g, 10.4 mL, 8.06 equiv) in CH_2Cl_2 (25 mL) was added slowly dropwise at -78 °C. After that, the mixture was allowed to warm to room temperature. Then the solvents were removed under reduced pressure at 40 °C. The residue containing the crude ketoaldehyde **2** was used in the next step without purification. Ketoaldehyde **2** was dissolved in anhydrous methanol (50 mL) and then powdered 3 Å molecular sieves (625 mg) were added. A solution of n-butylamine (2.02 g, 2.73 mL, 27.6 mmol) in anhydrous methanol (25 mL) was added, followed by the addition of 96% formic acid (1.32 g, 1.08 mL, 27.4 mmol), and sodium cyanoborohydride (1.45 g, 22.6 mmol, 2.5 equiv). *The pH should be maintained below 7 during this reaction. If needed, additional formic acid has to be added*. After the mixture was stirred at 50 °C overnight, 1.0 M NaOH solution was added until the pH was above 7. The mixture was filtered through Celite and the filtrate was diluted with water (50 mL), extracted twice with CH_2Cl_2 (100 mL) and dried over Na_2SO_4. Silica gel column chromatography, employing hexanes/ethyl acetate (80:20) furnished **3** as a pale yellow solid (3.2 g, 60% over two steps).

***N*-Butyl-1-deoxynojirimycin.** To intermediate **3** (3.20 g, 5.51 mmol) dissolved in methanol (50 mL) was added palladium chloride (665 mg, 3.75 mmol, 0.68 equiv). The reaction was stirred under hydrogen gas at 10 psi until the uptake of hydrogen stopped. The reaction mixture was filtered through Celite. The solvent was removed under reduced pressure and the residue was dissolved in a minimum amount of 30% aqueous methanol and loaded on to a Dowex 50Wx8 (mesh) ion exchange column (acid form). The column was eluted with water until the eluent tested negative for chloride

ions (dilute HNO_3/silver nitrate), and was then eluted with 1.0 M ammonium hydroxide. The ninhydrin positive fractions were combined and freeze-dried. Crystallization from dry methanol/dry acetone yielded 846 mg (70%) of the target compound. Mp = 129-130 °C Optical rotation $[\alpha]_D^{25}$ -15 (*c* = 0.93, H_2O). ^{1}H NMR (D_2O, 400 MHz): δ 3.91 (dd, *J* = 2.3, 12.8 Hz, 1H), 3.83 (dd, *J* = 2.7, 12.8 Hz, 1H), 3.54 (ddd, *J* = 4.9, 10.2, 14.3 Hz, 1H), 3.38 (d,d, *J* = 9.4 Hz, 1H), 3.25 (d,d, *J* = 9.3 Hz, 1H), 3.03 (dd, *J* = 5.0, 11.4 Hz, 1H), 2.74 (m, 1H), 2.60 (m, 1H), 2.30 (d, *J* = 11.1 Hz, 1H), 2.24 (dd, *J* = 2.7, 12.5 Hz, 1H), 1.46 (m, 2H), 1.28 (m, 2H), 0.90 (t, *J* = 7.3 Hz, 3H). ^{13}C NMR (100 MHz, D_2O): δ 74.7, 70.0, 66.7, 62.6, 60.2, 55.5, 52.1, 24.8, 20.0, 13.1; HRMS calcd for $C_{10}H_{22}NO_4$ $(M+1)^+$; found 220.1521.

Scheme 2. Synthesis of *N*-butyl-1-deoxygalactonojirimycin (*N*B-DGJ).

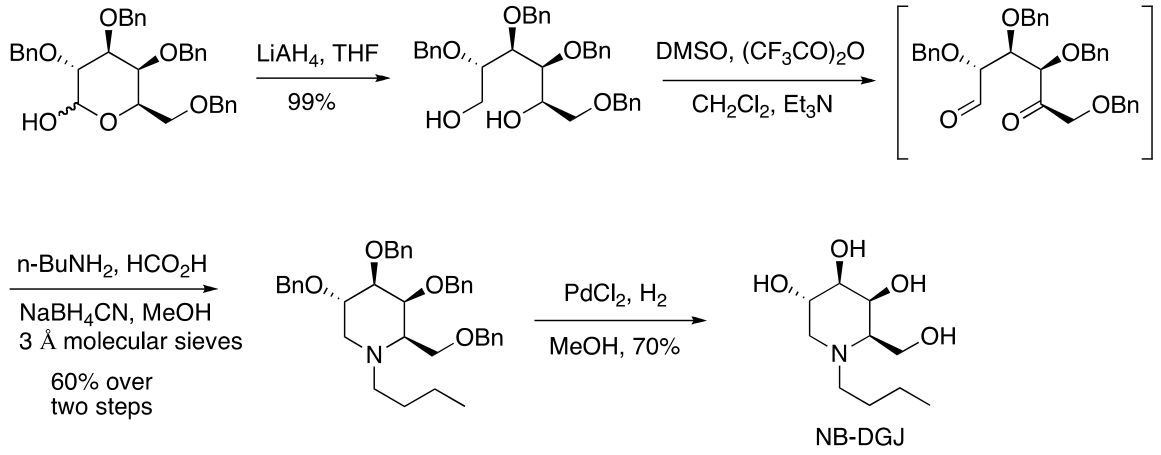

***N*-Butyl-1-deoxygalactonojirimycin (*N*B-DGJ).** *N*-Butyl-1-deoxygalactonojirimycin (*N*B-DGJ) was prepared using the method that was used for the synthesis of *N*B-DNJ. The starting material was 2,3,4,6-tetra-*O*-benzyl-D-glactonopyranose. The final product *N*-butyl-1-deoxygalactonojirimycin (*N*B-DGJ) was recrystallized from dry acetone. ^{1}H NMR (D_2O, 500 MHz): δ 4.07 (m, 1H), 3.87 (m, 1H), 3.84 (m, 1H), 3.78 (dd, *J* = 6.4, 11.5 Hz, 1H), 3.39 (dd, *J* = 3.2, 9.7 Hz, 1H), 3.03 (dd, *J* = 4.9, 11.4 Hz, 1H), 2.70 (m, 1H), 2.55 (m, 2H), 2.26 (t, *J* = 11.1 Hz, 1H), 1.46 (m, 2H), 1.27 (m, 2H), 0.89 (t, *J* = 7.3 Hz, 3H). HRMS calcd for $C_{10}H_{22}NO_4$ $(M+1)^+$; found 220.1575.

Analytical: The purities of *N*B-DNJ and *N*B-DGJ was determined by LC/MS and found to contain no impurities, using the following conditions:

Column: Acquity HSS T3 Column, 1.8 um, 2.1 x 30 mm
Solvent A: 10 mM ammonium acetate solution
Solvent B: 100% acetonitrile

Method:

Time	Flow rate	%A	%B
0 min	0.25mL/min	95%	5
1 min	0.25mL/min	50%	50
4 min	0.25mL/min	95%	5
5.5	0.25mL/min	95 %	5
6.5	0.25mL/min	95%	5

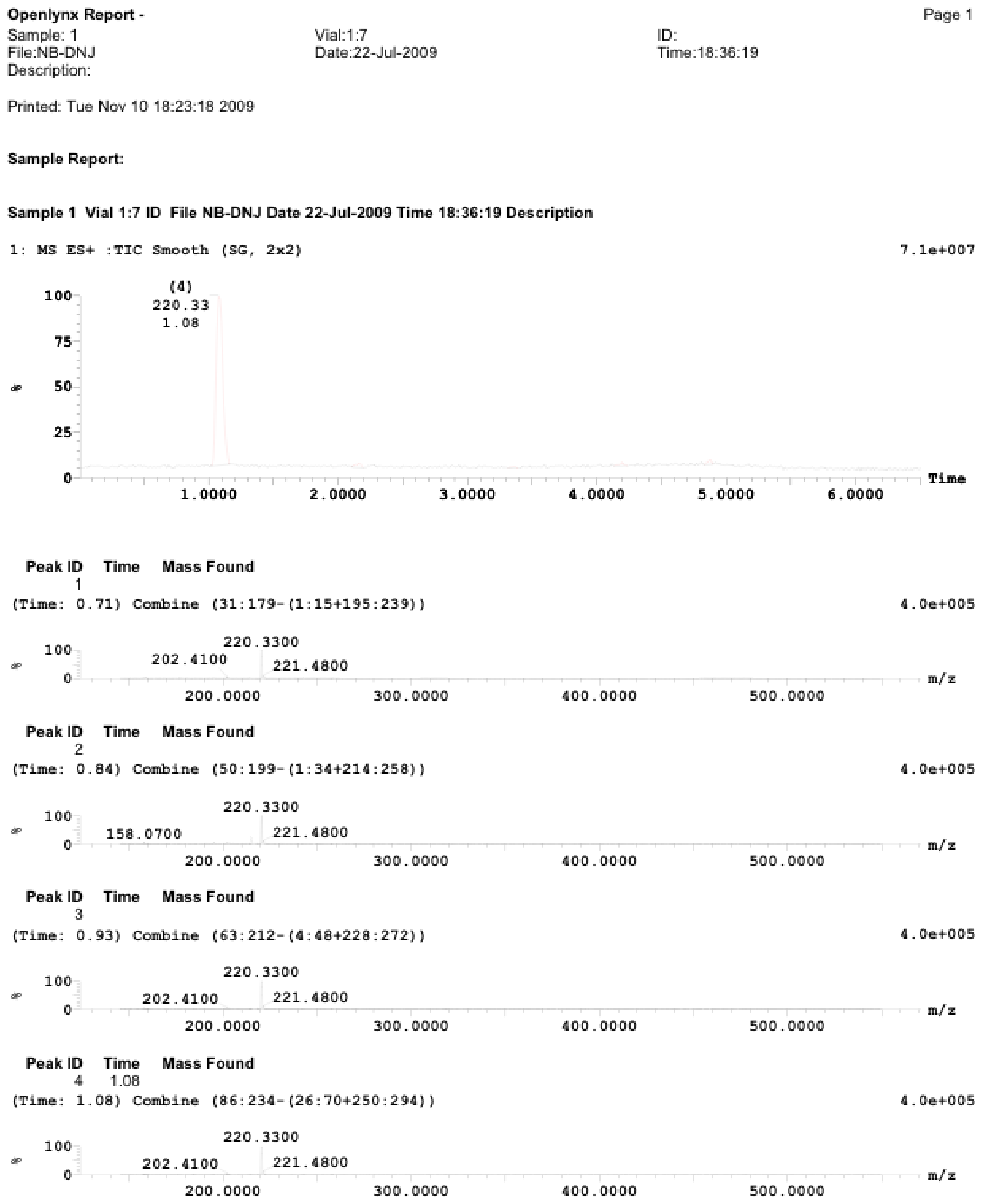

Fig. 1. LC/MS trace for *N*B-DNJ.

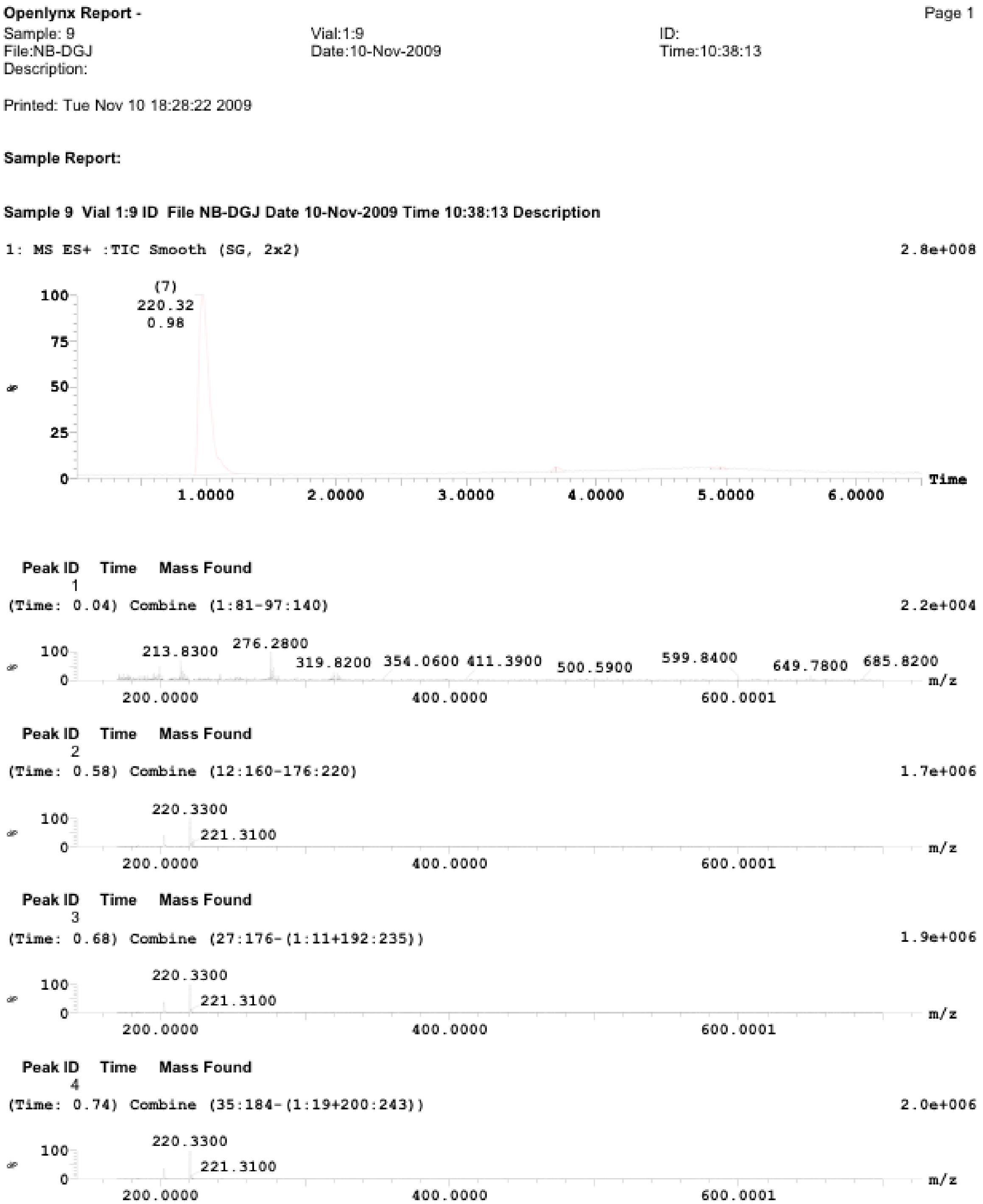

Fig. 2. LC/MS trace for *N*B-DGJ.

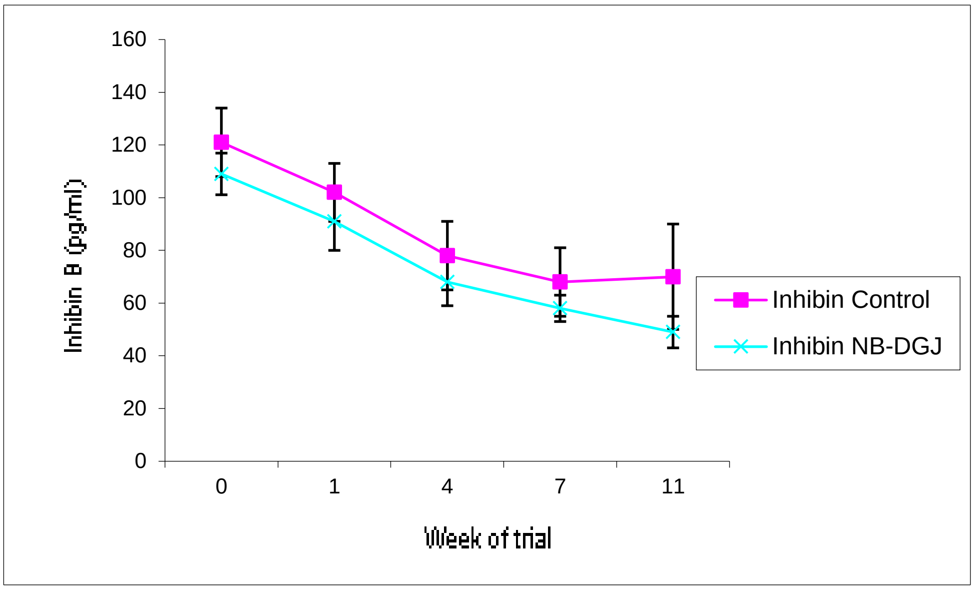

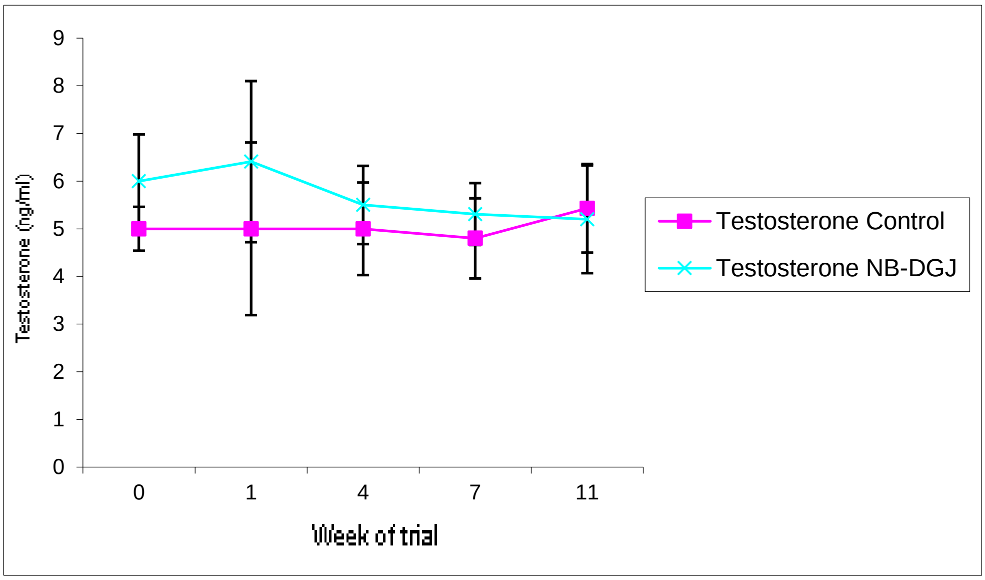

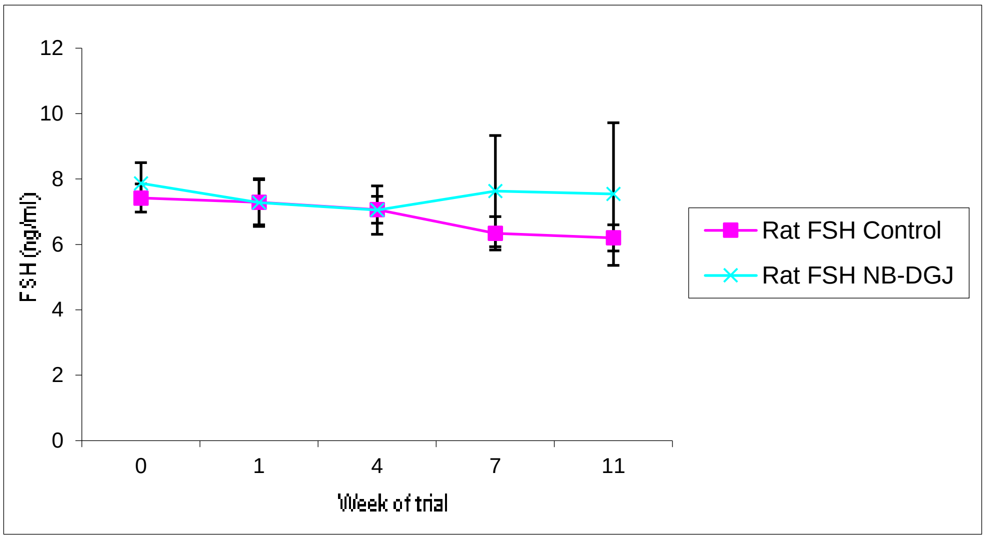

Fig. 3. Hormone levels in rats during *N*B-DGJ treatment schedule. The levels of inhibin, testosterone and rat FSH were similar in both control (vehicle) and *N*B-DGJ treated rats.

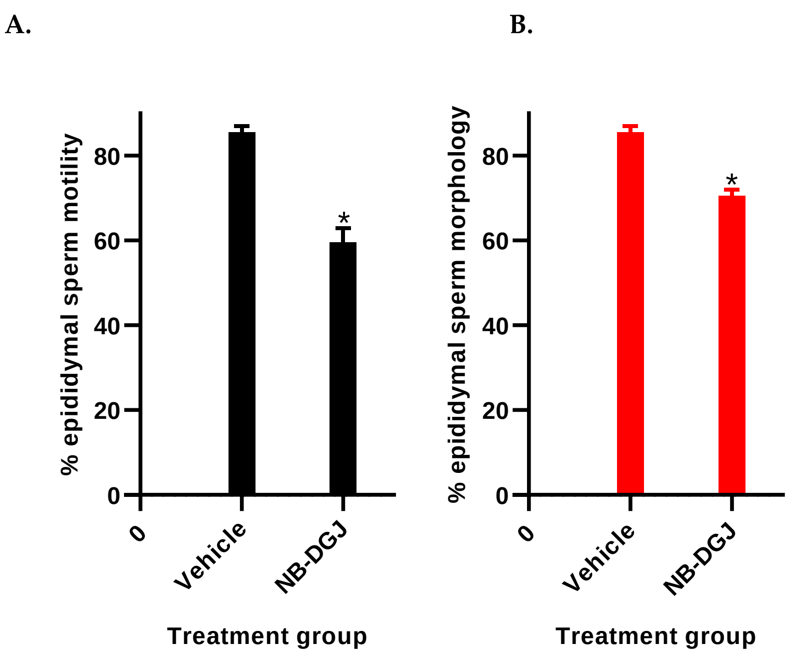

Fig. 4. Epididymal sperm motility (A) and sperm morphology (B) in vehicle and NB-DGJ treated rats at week 11 (completion of study) showed significant difference.

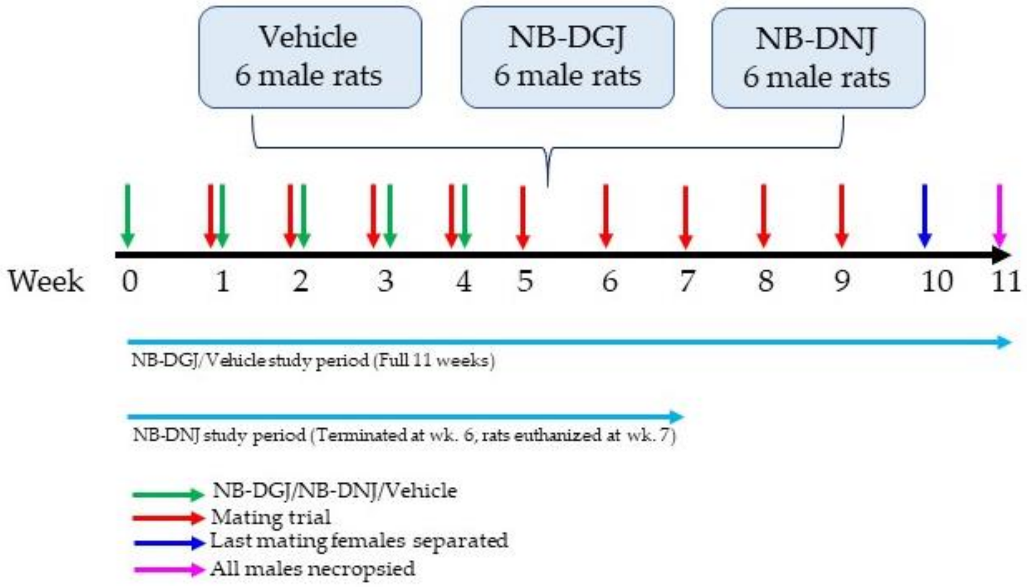

Fig. 5. Study design for rat mating trial

References:

1. Matos, C. R. R.; Lopes, R. S. C.; Lopes, C. C. Synthesis of 1-deoxynojirimycin and N-butyl-1-deoxynojirimycin. *Synthesis* 1999, 571-573.
2. Aman, F.; Lanz, M.; Scopes, D. I. C. Process for the production of deoxygalactonojirimycin derivatives. International patent WO 2004/054975.
